# High-flow nasal oxygen reduces the incidence of hypoxia in sedated hysteroscopy for assisted reproduction

**DOI:** 10.3389/fmed.2022.929096

**Published:** 2022-08-08

**Authors:** Ying Tang, Ping Huang, Di Chai, Xiao Zhang, Xiaoyi Zhang, Shaoyi Chen, Diansan Su, Yonglei Huang

**Affiliations:** Department of Anesthesiology, Renji Hospital, Shanghai Jiao Tong University School of Medicine, Shanghai, China

**Keywords:** hysteroscopy, *in vitro* fertilization, high-flow nasal oxygen, hypoxia, deep sedation, propofol

## Abstract

**Backgrounds and aims:**

Pain is the main reason for hysteroscopy failure. In day-surgical settings, hysteroscopy procedures are commonly performed with the patient under sedation. Hypoxia is the most common adverse event during sedation and can lead to severe adverse events. This study aimed to compare the incidence of hypoxia when using high-flow nasal oxygen (HFNO) with that when using regular nasal oxygen in patients undergoing hysteroscopy with sedation.

**Materials and methods:**

In this single-center, prospective, randomized, single-blinded study, 960 female patients undergoing elective diagnostic or operative hysteroscopy were randomly enrolled into the following two groups: the regular nasal group [O_2_ (3–6 L/min) covered by an HFNO] and the HFNO group [O_2_ (30–60 L/min)] from September 2021 to December 2021. All women were sedated with propofol (1.5 mg/kg) and remifentanil (1.5 μg/kg) in the operating room. The primary outcome was the incidence of hypoxia (75% ≤ SpO_2_ < 90%, < 60 s).

**Results:**

HFNO decreased the incidence of hypoxia (75% ≤ SpO_2_ < 90%, < 60 s), subclinical respiratory depression (90% ≤ SpO_2_ < 95%) and severe hypoxia (SpO_2_ < 75% for any duration or 75% ≤ SpO_2_ < 90% for ≥ 60 s) from 24.38 to 0.83%, from 11.25 to 1.46% and from 3.75 to 0%, respectively (*P* < 0.001).

**Conclusion:**

In procedures conducted to treat female infertility, HFNO can reduce hypoxia during hysteroscopy in patients sedated with propofol, and it can prevent the occurrence of subclinical respiratory depression and severe hypoxia.

## Introduction

Hysteroscopy can be used to intuitively and accurately detect the uterine cavity, observe its endometrium and treat cavity lesions while obtaining a diagnosis (1). Currently, hysteroscopy is recommended to be routinely conducted before an *in vitro* fertilization (IVF) cycle (2, 3). “See and treat” hysteroscopy has been advised as a first-line tool for screening uterine reproductive capacities in women experiencing infertility.

Although small or flexible hysteroscope is often conducted without any sedation or anesthesia, studies have reported that many patients are unable to endure the severe pain and hence do not complete the examination (4, 5). Pain is the main reason for hysteroscopy failure. In day-surgical settings, hysteroscopy procedures are commonly performed with sedation. The trend in inpatient hysteroscopy anesthesia has evolved from general anesthesia to sedation with intravenous anesthesia under monitored anesthesia care (6, 7). Propofol is widely used in the induction and maintenance of anesthesia, and remifentanil is a synthetic μ opioid often used to augment the propofol effect in intravenous anesthesia. These two common anesthetic agents have rapid onsets and offsets, which act synergistically to hypnotize and provide analgesia to patients with a rapid postoperative recovery of consciousness. Thus, intravenous anesthesia by propofol–remifentanil without intubation has been favored by anesthesiologists for short surgeries (8, 9). Paradoxically, respiratory depression, and even severe hypoxia, with these agents tend to occur in a dose-dependent manner. Prolonged hypoxia can lead to organic ischaemia, cardiac arrhythmia, permanent brain damage or even death, about which all anesthesiologists should be greatly concerned. The incidence of low SpO_2_ (<95%) during sedated hysteroscopy with hypnotic drugs has been reported to range from 3.3 to 51.2% (6, 7, 10, 11). However, there are no effective drugs or oxygen-supplying devices that can thoroughly eliminate hypoxia in sedated hysteroscopy.

High-flow nasal oxygen (HFNO) can provide patients with heated and humidified gas at an adjustable temperature (31–37°C), controllable flow (30–60 L/min) and regulated oxygen concentration (21–100%) via a nasal catheter. Through such high-flow modality, carbon dioxide can be expelled from the physiological dead space, and a positive airway pressure (3–7 cmH_2_O) can be provided to increase end-expiratory lung volume. These advantages enable this device to provide safe, comfortable and effective oxygen therapy in various clinical settings, as detailed in a recently published guideline (12). Many randomized controlled trials have shown that HFNO is the best non-invasive choice for supplying oxygen (13–17), especially in COVID-19 patients (13, 14). In these reports, Yilmazel et al. proved that HFNO was preferable during interventional bronchoscopy procedures and that patients had good compliance and tolerance (15). In a clinical study applying HFNO to esophagogastroduodenoscopy, Mazzeffi et al. reached the same conclusion (16). Lin et al. showed that HFNO could prevent hypoxia and severe hypoxia with very few adverse events and good tolerance during sedated gastroscopy (17). Inspired by these results, we designed this prospective randomized controlled trial and hypothesized that HFNO could provide effective and safe oxygen therapy during sedated hysteroscopy in women undergoing assisted reproduction.

## Materials and methods

This study was a single-center, single-blinded, prospective, and randomized clinical trial. The study was approved by the Renji Hospital Ethics Committee (No. KY2021-053-B) and registered at ClinicalTrials.gov (NCT05049395). We abided by the Consolidated Standards of Reporting Trials (CONSORT) statement.

### Patient population

We recruited female patients undergoing elective sedated hysteroscopy in the Reproductive Medical Center of Renji Hospital. The inclusion criteria were as follows: (1) age 20–50 years (20 years is the minimum legal childbearing age and 50 years is the maximum age for inclusion in infertility treatment), (2) American Society of Anesthesiologists (ASA) class I–II and (3) body mass index (BMI) ≤ 28 kg/m^2^. The exclusion criteria were as follows: (1) tendency of nasal mucosa bleeding, (2) history of cerebral diseases (e.g., cranial trauma and tumor), (3) history of diagnosed heart disease (e.g., heart failure, arrhythmia, and angina), (4) history of diagnosed pulmonary disease (e.g., upper respiratory tract infection, asthma and bronchitis), (5) severe liver and renal dysfunction, (6) oxygen dependency, (7) emergency surgery (e.g., multiple trauma), (8) pregnancy or positive pregnancy test and (9) cognitive dysfunction. All participants provided written informed consent. Consecutive participants were randomly (1:1) assigned to the regular nasal group or HFNO group.

### Hysteroscopy and anesthesia procedure

**FIGURE 1:**
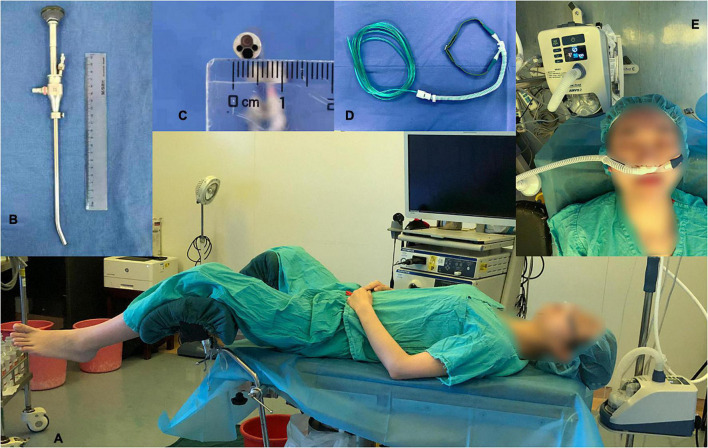
Surgical position, a hysteroscope and a high-flow nasal oxygen device. **(A)** Lithotomy position. **(B)** Outlook of the hysteroscope with a rigid 6.5-mm outer diameter and a 22° fore-oblique. **(C)** The 6.5-mm diameters of the hysteroscope. **(D)** Nasal cannula covered by the HFNO cannula. **(E)** High-flow nasal oxygen device and the parameters set in the study: adjustable temperature (37°C), gas flow (30–60 L/min) and oxygen concentration (100%).

All procedures were performed by a team of hysteroscopists with 10 years of working experience skilled in hysteroscopy and two assisting nurses who were their respective fixed working partners. The standard lithotomy position was required for surgery (Figure 1A). After anesthesia and sterilizing the surgical area, the speculum was tenderly placed into the vagina to stabilize the cervix, and the distension of the cervical canal was necessary to fit the diameter of the endoscope. Diagnostic or operative hysteroscopy was performed using a hysteroscopic instrument with a rigid 6.5-mm outer diameter and a 22° fore-oblique hysteroscope (patent number: CN88216856.8, ShenDa^®^, ShenYang, China; Figures 1B,C), which was connected to a visual system (Olympus, Japan). If the patient required treatment during the visualization of the uterine condition, biopsy graspers or scissors could pass through the hysteroscope. We used 0.9% normal saline as the distention medium, and cavity pressure was maintained by a pump at 80–100 mmHg.

All participants were residents in day-surgical wards, and no premedication was provided. After the peripheral intravenous line was opened, heart rate, blood pressure, and SpO_2_ were monitored routinely during the entire procedure. A regular nasal cannula looking seemingly like the HFNO nasal cannula (Figure 1D) or the HFNO nasal cannula (AIRVO 2 provided by Fisher and Paykel, Panmure, New Zealand, Figure 1E) was then inserted into the noses and oxygenation was started 1 min before anesthesia was initiated. All patients were anesthetized by the same anesthesiologist (one of the researchers) and sedated with a bolus of propofol (1.5 mg/kg) and remifentanil (1.5 μg/kg). After the patients lost consciousness, the oxygen flow rate was adjusted from 3 to 6 L/min in the regular nasal group and was increased from 30 to 60 L/min (37°C, 100% O_2_) in the HFNO group. The two specific oxygen flow rates were maintained until procedure completion. Sedation depth was assessed using the Ramsay Sedation Scale (RSS), and once the RSS score was > 4 (i.e., sluggish or no response to glabellar tap or loud auditory stimulus), the hysteroscopist inserted the speculum and began the surgery. The level of sedation was maintained at RSS > 4 throughout the procedure, and a single dose of propofol (0.2–0.5 mg/kg) was given when RSS was < 4 or when needed. The cannula was removed when the procedure was completed, and the side effects of HFNO and anesthesia were continually observed for at least 30 min in the postoperative care unit (PACU) in both groups.

### Outcome measures

We recorded the total dosage of propofol and remifentanil, vital signs (heart rate, blood pressure, and SpO_2_), the incidence of adverse sedation events as assessed by tools proposed by the World SIVA International Sedation Task Force (including respiratory depression, hypoxia, bradycardia and hypotension) (18) and the side effects of HFNO (including dry nose, nose pain, sore throat, headache, and barotrauma such as pneumothorax and subcutaneous emphysema). Meanwhile, treatments were implemented when the side effects of HFNO and sedative adverse events (decreased heart rate, decreased blood pressure, or hypoxia) occurred. The maneuvers to open the airway (jaw lifting, mask ventilation, laryngeal mask, or endotracheal intubation) were successively performed according to the actual clinical settings of hypoxia.

We strictly monitored the SpO_2_ of all the patients during the surgical period. The primary outcome was the incidence of hypoxia. Hypoxia was described as SpO_2_ falling to 75% ≤ SpO_2_ < 90% for < 60 s. The secondary outcomes were subclinical respiratory depression, severe hypoxia and other adverse events of sedation and HFNO. Subclinical respiratory depression was considered as SpO_2_ decreased to 90% ≤ SpO_2_ < 95%, whereas severe hypoxia was defined as either SpO_2_ < 75% or SpO_2_ < 90% for longer than 60s.

### Sample size calculation, blinding, and randomization

On the basis of the reported data of the average incidence of hypoxia, we used PASS software (version 16.0, NCSS, LLC, Kaysville, UT, United States) to estimate the sample size using the difference in hypoxia incidence (75% ≤ SpO_2_ < 90%, < 60 s) between the two groups. The incidence of hypoxia during sedated hysteroscopy was approximately 15%, which was expected to be reduced to 8% after oxygen inhalation with HFNO. With α = 0.05, power = 0.9 and factoring in a possible dropout rate of 10%, we calculated the sample size as 960 patients (480 in each group). All the patients were blinded in the study given that oxygen in the regular nasal group was supplied via a catheter covered by an HFNO nasal cannula. Moreover, to reduce the potential bias, an independent researcher blinded to the patient allocation was made in charge of follow-up in the PACU. A professional biostatistician, independent of data management and statistical analyses, generated the randomization sequence. The PROC PLAN program in SAS (version 9.0) was used to generate the randomization using a 1:1 allocation with block = 167 and length = 6. The results of the randomization were sealed in sequentially numbered envelopes.

### Statistical analysis

We performed statistical analysis using SPSS 26.0 (IBM, Armonk, NY, United States) according to the principle of intention-to-treat analysis. The Kolmogorov–Smirnov test was applied to test the normality of the individual variables. Normally distributed numerical data were expressed as mean ± SD, whereas skewed data were presented as the median [interquartile range (min–max)] or absolute numbers (proportion). The primary and secondary outcomes, namely, incidence of hypoxia and incidence of subclinical respiratory depression or severe hypoxia, were assessed by Wilcoxon rank-sum test. We analyzed numerical variables such as age, BMI, baseline SpO_2_, dosage of anesthetics, and procedure duration using an independent-samples Student *t* test. The minimum SpO_2_ in both groups was distributed non-normally and compared using the Mann–Whitney *U* test. Categorical variables such as ASA, Mallampati grade, snoring history, surgical approaches, maneuver of airway opening, adverse events of HFNO and sedation were compared using χ^2^ or Fisher exact test. *P* < 0.05 was considered statistically significant.

## Results

**FIGURE 2:**
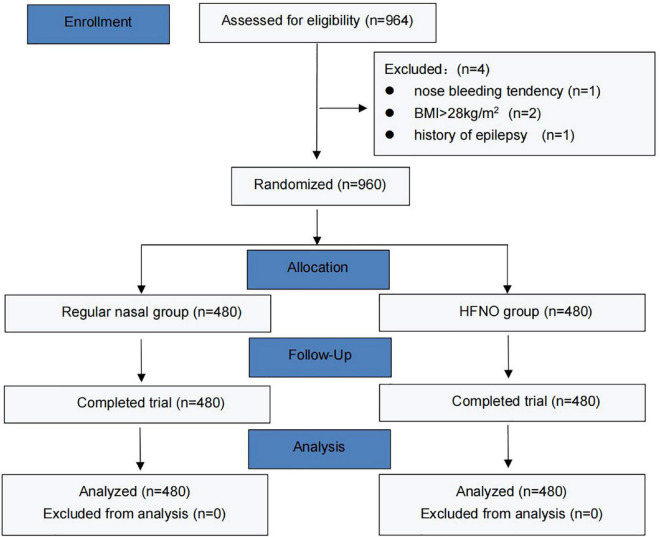
CONSORT flowchart of study. HFNO, high-flow nasal oxygen.

From September 2021 to December 2021, 964 patients were enrolled, 4 of whom were excluded (1 due to recent nose bleeding, 2 due to exceeded BMI and 1 due to a history of epilepsy). Finally, 960 patients were randomized into two groups, and their data were finally analyzed. Figure 2 shows the CONSORT flow diagram of the study. The general characteristics of the patients, including age, weight, height, BMI, ASA status or Mallampati grade, snoring history and baseline SpO_2_ did no differ between the two groups (Table 1). Table 2 lists the duration of the procedure and total dosage of propofol and remifentanil, which were not significantly different between the two groups.

**TABLE 1:** General characteristics of the patients undergoing elective diagnostic or operative hysteroscopy.

	Regular nasal group (*n* = 480)	HFNO group (*n* = 480)
Age, years	33.0 (4.3)	33.2 (5.0)
Weight, kg	56.9 (7.9)	56.9 (7.5)
Height, cm	161.0 (5.2)	161.0 (4.3)
BMI, kg/m^2^	21.9 (2.6)	21.9 (2.5)
ASA grade I/II	480/0 (100%)	480/0 (100%)
Mallampati I/II	478/2 (99.6%/0.4%)	478/2 (99.6%/0.4%)
Snoring history	2 (0.4%)	1 (0.4%)
Baseline SpO_2_,%	98.9 (0.5)	98.9 (0.4)

Values are mean (SD) or number (proportion). HFNO, high-flow nasal oxygen; BMI, body mass index.

**TABLE 2:** Data of sedated hysteroscopy procedure.

	Regular nasal group (*n* = 480)	HFNO group (*n* = 480)	*P*-value
Duration of procedure, min	5.3 (2.9)	5.2 (2.8)	0.522
Total dosage of propofol, mg	92.5 (17.7)	91.64 (17.2)	0.422
Total dosage of remifentanil, μg	85.2 (11.6)	85.19 (11.1)	0.945
Hysteroscopic approaches			
Diagnosis	357 (74.4%)	358 (74.6%)	1.000
Diagnosis and operation	123 (25.6%)	122 (25.4%)	1.000

Values are mean (SD) or number (proportion). HFNO, high-flow nasal oxygen.

### Primary outcome

**FIGURE 3:**
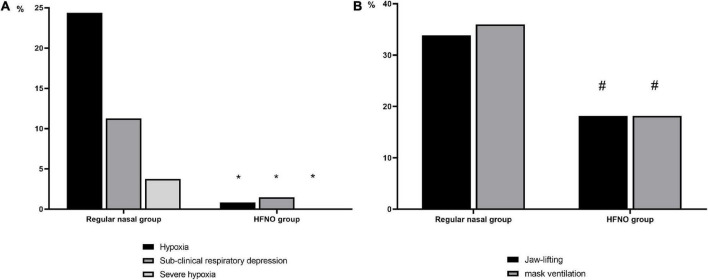
Incidence of hypoxia events and their interventions. **(A)** Incidence of hypoxia events in both groups. Compared with the regular nasal group, HFNO significantly decreased the incidence of hypoxia (75% ≤ SpO2 < 90%, < 60 s), subclinical respiratory depression (90% ≤ SpO_2_ < 95%) and severe hypoxia (SpO_2_ < 75% for any duration or 75% ≤ SpO_2_ < 90% for ≥ 60 s) from 24.38 to 0.83%, from 11.25 to 1.46% and from 3.75 to 0% (*P < 0.001, respectively). **(B)** Interventions during hypoxia. Compared with the regular nasal group, lower proportion in the HFNO group required oxygen improvement by jaw lifting or mask ventilation. In the regular nasal group, 132 patients needed their airway opened to relieve hypoxia, accounting for approximately 70% (69.84%) of the 189 patients; of these, 64 (33.86%) required jaw lifting and 68 (35.98%) required mask ventilation. However, in the HFNO group, only two patients required jaw lifting and two needed mask ventilation, which, respectively, accounted for 18.18% (^#^*P* < 0.001, respectively). No patients were intubated in either group.

In the regular nasal group, 117 of 480 patients developed hypoxia, whereas in the HFNO group, hypoxia occurred in only 4 of 480 patients. Figure 3A shows that HFNO significantly decreased the incidence of hypoxia from 24.38 to 0.83% (*P* < 0.001).

### Secondary outcome

**FIGURE 4:**
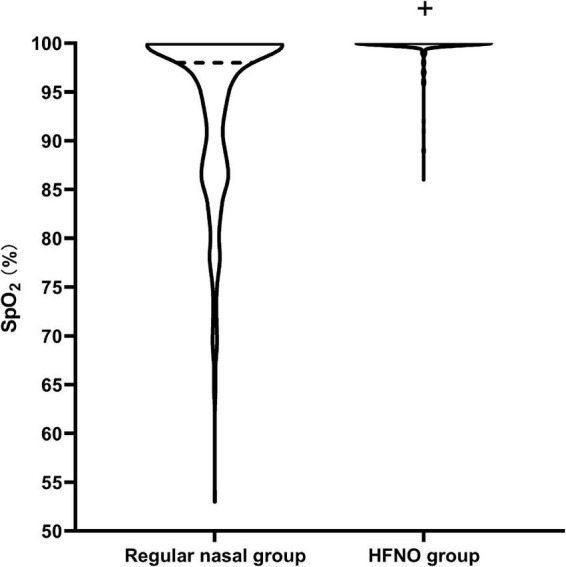
Diagram of the minimum SpO_2_ during the procedure. The violin plot visually represents the distribution of minimum SpO_2_. The minimum SpO_2_ during the procedure was 98% [12% (53–100%)] in the regular nasal group and 100% [0% (86–100%)] in the HFNO group (^+^*P* < 0.001). In the regular nasal group, the dark dotted line represents the medium SpO_2_ and is located at 98%, representing that most patients could maintain SpO_2_ at 98%. The lowest dotted line at 53% indicates there had one patient who experienced severe hypoxia in the study. Almost all patients in the HFNO group were able to maintain an oxygenation level of 100%, and patients seldom experienced hypoxia. No patients in the HFNO group experienced severe hypoxia.

A total of 189 patients in the regular nasal group experienced different hypoxia levels; among these, 18 patients developed severe hypoxia. Of the 189 patients, 132 (69.84%) required airway opening to relieve hypoxia, of whom 64 (33.86%) needed jaw lifting and 68 (35.98%) needed mask ventilation. In the HFNO group, seven patients experienced subclinical respiratory depression and four patients had hypoxia; no patients experienced severe hypoxia. Meanwhile, only 4 of 11 patients (36.36%) required jaw lifting or mask ventilation (*P* < 0.001, Figure 3B). No patients were intubated in either group. During the procedure in the regular nasal group, the minimum SpO_2_ was 98% [12% (53–100%)]; in the HFNO group, it was 100% [0% (86–100%)] (*P* < 0.001; Figure 4).

There was no statistically significant difference in the paradoxical responses of hysteroscopic surgery and dilation of the cervix between the two groups (*P* > 0.05; Table 3). HFNO-related adverse events were exactly slight (Table 3). Dry nose and rhinalgia were the most common symptoms after anesthesia recovery. Only seven patients complained of dry nose, which resolved 30 min after anesthesia recovery. No other adverse events were observed. Table 3 lists the incidence of postoperative pain and occurrence of hypotension and bradycardia. Only a small proportion of patients in both groups experienced mild menstrual-like pain (visual analogue scale 3–4) after hysteroscopy, and the occurrence of hypotension and bradycardia was under control.

**TABLE 3:** The adverse events of hysteroscopy, HFNO, and sedation.

	Regular nasal group (*n* = 480)	HFNO group (*n* = 480)	*P*-value
Adverse events of hysteroscopy			
Cervix dilation reaction	11 (2.3%)	8 (1.7%)	0.487
Body movement	1 (0.4%)	2 (0.4%)	1.000
Postoperative pain*	12 (2.5%)	15 (3.1%)	0.558
Adverse events of HFNO			
Dry nose	54 (11.3%)	7 (1.5%)	<0.001
Other adverse^†^	0	0	NS
Sedative events			
Bradycardia	27 (5.6%)	16 (3.3%)	0.086
Hypotension	1 (0.4%)	0	1.000

Values are number (proportion). HFNO, high-flow nasal oxygen; NS, there is no need to compare.

*Postoperative pain refers to mild menstrual-like pain (visual analogue scale 3–4) after hysteroscopy according to the description of patients.

^†^Other adverse refers to nose pain, sore throat, headache and barotrauma such as pneumothorax, subcutaneous emphysema.

## Discussion

Our study is the first to apply HFNO for deep sedation in women undergoing hysteroscopy for IVF. HFNO can reduce the incidence of hypoxia and can prevent the occurrence of subclinical respiratory depression and severe hypoxia.

There is no international consensus regarding which anesthesia regimen is optimal for hysteroscopy (19). The invention of small and flexible hysteroscopes has reduced the need for anesthesia and has enabled hysteroscopy to be completed with local anesthesia in an outpatient setting (20–22). However, it should be remembered that pain tolerance in office hysteroscopy depends on the skill of the operator performing the paracervical block or topical anesthesia. Even with small size lenses or soft microscopy for outpatient hysteroscopy, moderate to severe pain is inevitable in some patients (4, 5). In this way, day-surgical inpatient hysteroscopy with the participation of an anesthesiologist is common in reproductive centers in China, and it has the advantage of sedation and analgesia. Moreover, special pathologies still require treatment in the operation room. Under anesthesia, thicker lenses (outer diameter > 5 mm) can pass through the cervix after cervical dilation to enable a wider and clearer visualization of the uterus. Furthermore, cervical dilation might facilitate embryo transfer and the introduction of insemination catheters 31 days before embryo transfer (23). In addition, considering the possible anxiety women experience during IVF cycles (24) and the hysteroscopists’ satisfaction with no movements under deep sedation, hysteroscopy under sedation with propofol and remifentanil is the preferred choice of anesthesia, and it was shown in early 2008 to be safe and effective during hysteroscopy (7).

Hypoxic adverse events in intravenous anesthesia are very common, if they are undetected or not treated in time, hypoxia may lead to unimaginable consequences, such as arrhythmia, permanent brain damage and even death. Once hypoventilation is noticed—whether in the form of decreased respiratory rate, shallow chest wall motion or monitoring abnormality—the physician must assess the seriousness and risk of deterioration (25).

In the review of the literature on sedated endoscopic examination, anesthesiologists seem to be more interested in hypoxia research in sedated gastroenteroscopy. Previous articles reported that supplemental oxygenation could be offered with close monitoring of anesthesiologists through diverse preventive or remedial approaches, such as endoscopic mask (26), supraglottic jet oxygenation (27), Wei Jet nasal (Well Lead Medical Co., Guangzhou City, China) (28) or jaw lifting and pressurized mask ventilation. Despite rigorous monitoring and the development of new tools to help in maintaining oxygenation, respiratory desaturation or hypoxia still occurs during procedures conducted under sedation. In our study, approximately 28% of the patients experienced hypoxia, which was higher than that in sedated gastroscopy (approximately 6–15%) (26–28). A comparison of hypoxic occurrence between the two different types of endoscopy is meaningless, but hypoxia in sedated hysteroscopy requires greater research attention, and a new method should be introduced in clinical practice to improve this phenomenon.

In our study, HFNO decreased the incidence of hypoxia in sedated hysteroscopy. We speculated that the mechanisms were the same as reported previously by Lin et al., namely, the continuous positive airway pressure and a high fraction of inspired oxygen (FiO_2_) offered by the HFNO device (17). Their results excited us that HFNO could reduce the incidence of hypoxia to 0% during sedated endoscopy. Imperfectly, hypoxia still existed in our study, but the incidence was obviously lower (0.83%), and there were no cases with severe hypoxia. We analyzed why HFNO could not lower the proportion of hypoxia to 0% in our study. This might be related primarily to the characteristics of hysteroscopy itself and the medication. First, surgical stimulation in hysteroscopy is stronger than that in gastroendoscopy mainly because of the dilation of the cervix and uterine cavity surgery (1), which can induce unbearable pain and abortion syndrome reactions (vagus nerve excitation) such as a pale face, sweating, nausea, vomiting and bradycardia. When the vagus nerve system dominates, the patient’s breathing becomes slow and shallow, and even apnoea can occur (29). In our study, approximately 10 cases of cervix dilation reaction and body movement appeared in both groups because of pain, and low-oxygen ventilation might be started at that time. Unfortunately, we did not monitor respiratory frequency or end-tidal carbon dioxide (EtCO_2_). Second, patients were placed in the lithotomy position. Under anesthesia, this special surgical position alters physiological respiratory function, lifts the diaphragm and reduces lung compliance and functional residual capacity, followed by low oxygenation (30). In addition, it is believed that remifentanil, particularly when combined with propofol, is an important cause of intraoperative respiratory depression (31). These factors seemed to be highly related to the occurrence of hypoxia during hysteroscopy.

In addition to HFNO, there have been attempts to use a new airway tool for sedated hysteroscopy. Liang et al. compared new methods of supraglottic jet oxygenation and ventilation (SJOV), WEI Nasal Jet tube (WNJ) and mask oxygen for obese patients under intravenous anesthesia during hysteroscopy (32). They conducted a pairwise comparison of the three methods, with the primary outcome being the incidence of pressure of end-tidal carbon dioxide (PetCO_2_) < 10 mmHg or SpO_2_ < 95%. These authors found that, compared with the control group, the incidence of PetCO_2_ < 10 mmHg or SpO_2_ < 95% in the SJOV group dropped from 36 to 9% (*P* = 0.009) or from 33 to 6% (*P* = 0.006), respectively. Compared with the WNJ, the use of SJOV significantly decreased the incidence of PetCO_2_ < 10 mmHg or SpO_2_ < 95% from 33 to 9% (*P* = 0.017) or from 27 to 6% (*P* = 0.023), respectively. From the disparate results, we cannot easily conclude which device, HFNO or SJOV, is more helpful for reducing hypoxia during hysteroscopy because the two studies were conducted with different protocols. We used the nasal cannula as the control pattern, whereas Liang et al. used mask ventilation. Thus, the incidence of SpO_2_ < 95% was different between the two control groups. Our study population did not include obese patients, who were more likely to experience airway obstruction under anesthesia. In addition, we administered a single bolus induction with propofol (1.5 mg/kg) and remifentanil (1.5 μg/kg) and maintenance of single bolus propofol when needed, ensuring RAS > 4. In the study by Liang et al., the anesthesia protocol was a bolus injection with propofol (1.5–2.0 mg/kg) and remifentanil (0.5 μg/kg) for induction and a continuous injection of propofol (3–5 mg/kg/h) and remifentanil (0.05–0.08 μg/kg/h) for maintenance. As is well known, responsiveness is always suppressed as the level of sedation increases, as does the potential ability of patients to control their airways, ventilation and cardiovascular function.

We designed a remedial intervention when low-oxygen saturation was difficult to correct in both groups. In the control group, 132 patients (64 for jaw lifting and 68 for mask ventilation) experienced the remedial intervention, consisting of 69.84% with low oxygenation (SpO_2_ < 95%), whereas in the HFNO group, only 2 patients received jaw lifting and 2 patients were ventilated by mask, comprising 36.36% of 11 patients with low oxygenation (SpO_2_ < 95%). Accordingly, in most patients, relief of inadvertent oxygen depressant with deep anesthesia when using HFNO and effective autonomous breathing will resume before the onset of any clinically worse oxygen desaturation (25). Theoretically, HFNO seldom has side effects. In our study, seven patients experienced dry nose in the HFNO group, which was far fewer than that with the normal oxygen absorption method (HFNO vs. regular nasal cannula, 7 vs. 54, 1.5 vs. 11.3%).

Oxygen toxicity usually occurs during oxygen inhalation at high concentrations over a prolonged time (33, 34). In our study, the duration of the procedure was very short (mean duration time was approximately 5 min in both groups, see Table 2) and we did not observe lung injuries caused by oxygen toxicity after surgery during this period, as reported in other clinical HFNO studies (17, 35). Fortunately, in the actual clinical practice, the flow rate or the oxygen concentration of HFNO can be adjusted according to the patients’ situation to avoid the occurrence of oxygen toxicity.

Future research should seek to resolve some of the limitations of this study. First, this was a single-blinded clinical study, which might cause potential bias. Second, medications or methods used for sedation vary among different institutions and different countries. Fortunately, the intravenous anesthetics that we selected are widely used because of their clinical compatibility, and the data obtained in our study could be representative in most clinical settings. Third, a large proportion of obese women with polycystic ovary syndrome experienced infertility, and a more susceptible population was excluded from this study. Fourth, the procedure duration in our study was relatively short. Thus, anesthesia was maintained with a single additional dose of propofol when needed. We did not investigate HFNO performance during long-time surgery with more maintained anesthetics. Therefore, further HFNO research in hysteroscopy should be performed.

## Conclusion

A large population of women experiencing infertility require hysteroscopy with deep sedation. Precautions should be taken to maintain ventilation during sedated hysteroscopy. HFNO can reduce the incidence of hypoxia in patients receiving deep sedation. Thus, HFNO is recommended for women undergoing sedated hysteroscopy during an IVF cycle.

## Data availability statement

The raw data supporting the conclusions of this article will be made available by the authors, without undue reservation.

## Ethics statement

The studies involving human participants were reviewed and approved by Renji Hospital Ethics Committee. The patients/participants provided their written informed consent to participate in this study. Written informed consent was obtained from the individual(s) for the publication of any potentially identifiable images or data included in this article.

## Author contributions

DS and YH: study conception. YT, PH, DS, and YH: study design. YT, PH, XYZ, SC, DS, and YH: data analysis. YT, PH, DC, XZ, DS, and YH: data interpretation. YT, PH, YH, and DS: drafting of the manuscript. All authors study conduct, critical revision of the manuscript for important intellectual content, and agreed to be accountable for all aspects of the work in ensuring that questions related to the accuracy or integrity of any part of the work are appropriately investigated and resolved.

## Abbreviations

HFNO: high-flow nasal oxygen
IVF: *in vitro* fertilization
COVID-19: Corona Virus Disease 2019
ASA: American Society of Anesthesiologists
BMI: body mass index
RSS: Ramsay Sedation Scale
SpO_2_: pulse oximetry or pulse oxygen saturation
SD: standard deviation
PACU: postoperative care unit
CONSORT: Consolidated Standards of Reporting Trials
FiO_2_: fraction of inspired oxygen
EtCO_2_: end-tidal carbon dioxide
SJOV: supraglottic jet oxygenation and ventilation
WNJ: WEI Nasal Jet tube
PetCO_2_: pressure of end-tidal carbon dioxide.
